# Does Overnight Culture of Cleaved Embryos Improve Pregnancy Rate in Vitrified-Warmed Embryo Transfer Programme?

**DOI:** 10.21315/mjms2019.26.2.6

**Published:** 2019-04-30

**Authors:** Azam Agha-Rahimi, Marjan Omidi, Fatemeh Akyash, Azita Faramarzi, Forough Alsadat Farshchi

**Affiliations:** 1 Research and Clinical Center for Infertility, Yazd Reproductive Sciences Institute, Shahid Sadoughi University of Medical Science, Yazd, Iran; 2 Stem Cell Biology Research Center, Yazd Reproductive Sciences Institute, Shahid Sadoughi University of Medical Science, Yazd, Iran; 3 Fertility and Infertility Research Center, Health Technology Institute, Kermanshah University of Medical Sciences, Kermanshah, Iran

**Keywords:** vitrification, overnight culture, pregnancy, live birth

## Abstract

**Background:**

Vitrification is a routine procedure in assisted reproductive technique (ART) lab. However, there is widespread variability between protocols of different centres. The aim of this study was to compare the chemical pregnancy, clinical pregnancy and live birth rates between one-day embryo culture and immediate transfer for frozen-thawed embryo transfer (FET) cycles.

**Methods:**

In this cohort retrospective study, 366 FET cycles were divided into two groups: Group A, the embryos were warmed one day before transfer, and were cultured overnight; Group B, the embryos were warmed on the same day of transfer, at least were cultured 1 h before embryo transfer (ET). Chemical and clinical pregnancy and live birth rates were compared between two groups.

**Results:**

The chemical pregnancy was higher in group A than B (37.9% versus 28.9%), but this difference was not significant (*P* = 0.07). Clinical pregnancy (30.8% versus 24.1%) and live birth (19.8% versus 22.05%) were similar in group A and B, (*P* = 0.15), and (*P* = 0.8). *Conclusion*: In conclusion, overnight culture and confirmation of mitosis resumption was not essential for FET cycles in vitrification method.

## Introduction

### Background

Cryopreservation of surplus embryos after fresh embryo transfer (ET), donation programme, embryo storage for fertility preservation, and embryo freezing in patients at risk of ovarian hyperstimulation syndrome (OHSS) is a routine procedure in all ART labs (1). Roque et al. in a meta-analysis concluded that frozen-thawed embryo transfer (FET) has better outcome compared fresh ET regarding pregnancy and live birth rates. This explains the better synchronization between embryo and endometrium cause these results (2). It was reported that morphological and molecular changes to the endometrium occurred after controlled ovarian hyperstimulation (COH) and reduced endometrial receptivity. Therefore, FET reduces the risk of OHSS and improves outcomes for both the mother and baby (3). Also, Wu et al. reported that FET cycles had better clinical outcomes compared to fresh and even blastocyst transfer in normal responder women (4). But, Basirat et al. showed that the pregnancy rate did not differ significantly in ET and FET (5).

The success rate of FET cycles depends on several factors. Chi and associates demonstrated that 3 days embryos have higher clinical pregnancy rates in poor responders compared to 2 days embryos, although, these results were not different in normal responders (6). Endometrial preparation is another key factor that effect on FET results (7, 8). The two most popular methods to select frozen-thawed embryos are based on the post thawed duration culture. According to observation of survive of blastomeres after warming in short culture and observation of proliferation of blastomeres in long overnight culture. But it is not clear which methods is better (9). There are two protocols about culture of vitrified embryo after warming (10). Some centres do FET in the same day of warming and other centres transfer the warmed embryos after 24 h culture. It seems there are no studies that compare these strategies regarding to pregnancy outcome in vitrification method.

### Objectives

The aim of this study was compare the chemical and clinical pregnancy, and live birth rates in FET cycles after one day embryo culture or immediate ET in the same day of warming.

## Method and Materials

### Setting

This retrospective cohort study has been done in the Yazd Reproductive Sciences Institute. Patients underwent FET cycles were evaluated between January 2015 and June 2016.

### Study Design and Study Size

A total of 366 FET cycles were divided into two groups; groups A and B. Group A (*n* = 195) FET cycles which their embryos were warmed one day before ET and were cultured overnight. Group B (*n* = 166) included cycles which their embryos were warmed on the day of ET, with at least 1 h culture before ET.

### Participant

Inclusion criteria included cycles with embryos were vitrified in 2 days. Exclusion criteria included the cycles with damaged embryos, gamete or embryo donation, embryos that generated from cryopreserved oocyte and sperm. The demographic characteristics, number of transferred embryos, the quality of embryos, chemical and clinical pregnancy, and live birth rates in each cycle were recorded and compared between two groups. This work was approved by the ethics committee of our institute in Yazd, Iran (code of ethics: IR.SSU.RSI.REC.1396.10). All patients have signed up the satisfaction form before the intervention.

### Vitrification and Warming

Vitrification and warming was done using vitrolife Kit (Vitrolife, Kungsbacka, Sweden) according to its instructions. Briefly, for vitrification, the 2 days embryos from culture dish transferred into the Vitri 1™ Cleave and for at least 5 min. Then the embryos exposed to Vit 2™ Cleave for 2 min. Finally the embryos transferred to Vit 3™ for 30 s and loaded on the Cryotop or Cryothech and submerged in liquid nitrogen. All manipulations of the embryos were carried out at 37 °C (on a heated stage). For warming, all manipulations of the embryos were carried out at 37 °C (on a heated stage). The vitrified embryos quickly placed into Warm 1™ Cleave for 10–30 s, 1 min in Warm 2™ Cleave, 2 min in Warm 3™ Cleave and 5 min in Warm 4™ Cleave. Then the embryos transferred to culture medium and cultured in 37 °C and 5% CO_2_.

### Embryo Grading

The embryos were categorised to grade A, with regular size blastomere, and no fragmentation; grade B, the embryos with uneven blastomeres and/or less than 10% fragmentation; grade C, the embryos with more than 10% fragmentation but no more than 25% of blastomeres. Embryos with grades A and B were considered as top quality embryos (11).

### Preparation of the Endometrium

The artificial cycles began by taking oral estradiol valerate (2 mg, Aburaihan Co., Tehran, Iran) 6 mg daily from the second day of the menstrual cycle. Ultrasonography was performed on day 12–13 of the cycle. Endometrial thickness was measured at its thickest part in the longitudinal axis of the uterus. When the endometrial thickness was ≥ 8 mm, the vaginal progesterone (Cyclogest; Actavis, UK limited, England) was started 400 mg twice daily and oral estradiol was continued.

### Vitrified-Warmed ET

ET was carried out after 3 days of progesterone receiving. A maximum of 2 vitrified-warmed embryos were transferred under ultrasound guidance using by a Cook® Sydney IVF (Cook Medical, USA). Estradiol valerate and progesterone supplementations were continued for 2 weeks after ET, and if the serum beta human chorionic gonadotropin (βHCG) was positive, hormone supplementations were continued until 12 weeks of gestation.

### Assessment of Pregnancy Outcome

Serum β HCG levels were measured 14 days following ET. If the test was positive, the chemical pregnancy was recorded. An ultrasound scan was carried out 4 weeks after ET. The observation of at least one sac with heart beat considered positive clinical pregnancy. Live birth rate was considered the number of deliveries that caused a live baby in at 100 FET cycles.

### Statistical Methods

We used SPSS software (SPSS version 20, Chicago, IL) for statistical analysis. Independent sample *t*-test was used for comparison of mean values and chi-squared test was used for comparison of percentages. Logistic regression was used for evaluating effects of different parameters on chemical and clinical outcomes. *P*-value *<* 0.05 was considered to be statistically significant.

## Results

As shown in Table 1, demographic characteristics including age and cause of infertility were not significantly different between the two groups. In addition, number of transferred embryos and the quality of embryos was not different in two groups.

Pregnancy outcomes were presented in Table 2. Chemical pregnancy was 28.9% in group A and 37.9% in group B with *P*-value of 0.07. Clinical pregnancy rate was not significant in groups A and B (24.1 versus 30.8, *P* = 0.1). In addition, the live birth was similar in both groups.

In another view, we compared the FET outcome in different grades of embryo. In total of 361 FET cycle, 61 cycles (16.3%) had grade A embryo, 255 cycle (70.6%) had grade B embryo and 45 cycle (12.5%) had grade C embryos. There were no predictive factors for chemical and clinical pregnancy (Table 3).

## Discussion

Our study was evaluated the effect of overnight culture in FET cycles in vitrification method. In our centre, the embryos were routinely warmed one day before ET. Only embryos with further cleavage were transferred and if in a cycle after overnight culture, we did not observe any more cleavage, another cryotop was warmed in day of ET. In the days that the previous day was in the weekend, embryo warming was done on the same day of ET. In this study, we observed that rarely vitrification and warming cause arrested development in high quality embryo and about of 90% of warmed embryos had sign of cleavage resumption after overnight culture (unpublished data). We followed up the results of embryo warming in day of transfer or in the previous day. Although chemical pregnancy was higher in overnight culture group with difference of about 10%, but live birth rate that is the final result was similar between groups. This result showed despite the reports about slow freezing, in vitrification method, overnight culture and check of resumption of mitosis is not essential. Tang et al. reported that mitosis resumption after overnight culture is a critical factor for improved pregnancy rate in FET cycles in slow freezing method (12). Solé et al. confirmed a direct correlation between the degree of development of the embryos after overnight culture and their implantation potential. They reported that the embryos with at least two blastomeres cleaved, had significant more implantation rate in slow freezing cycles (13). Furthermore, it was concluded that the embryos were not cleaved after overnight culture had very low pregnancy rates. Also, it was reported that transfer of frozen-thawed embryos with or without overnight culture after thawing was not different pregnancy rate (14). Although, all of these reports were related to slow freezing protocol. There are several reports about the factor of resumption of mitosis after vitrification on pregnancy rate, but to our knowledge, this is the first study evaluating the effect of overnight culture of warmed embryos on live birth result. In this study, we observed that rarely vitrification and warming cause arrested development in high quality embryo and about of 90% of warmed embryos had sign of cleavage resumption after overnight culture (unpublished data). We followed up the results of embryo warming in day of transfer or in the previous day. Although chemical pregnancy was higher in overnight culture group with difference of about 10%, but live birth rate that is the final result was similar between groups. This result showed despite the reports about slow freezing, in vitrification method, overnight culture and check of resumption of mitosis is not essential. Van Landuyt et al. reported that in 35 FET cycles that embryos arrested after overnight culture in slow freezing and vitrification, none of them led to ongoing pregnancy (15). Also, they reported that further cleavage is a critical factor for embryos with damaged blastomers. Because these embryos had a lower potential overnight development, but if these cleave further after warming, it was not influence on implantation rate (15). Gallardo et al. reported that approximately 90% of day 3 warmed embryos had cleavage division after overnight culture. They concluded that day 3 embryos with ≤ 6 cells after overnight culture had very low implantation rate chance (≤ 1%) (16). Chi et al. discussed about prolonged culture before cryopreservation. They concluded that this strategy could increase the possibility of obtaining more high quality with better developmental potential for vitrification. Their results showed that day 3 embryos vitrification did not affect survival rate, but yielded better clinical outcomes compared to day 2 embryos, especially in poor responder patients (6). Meng et al. expressed warmed day 3 embryos that compacted after overnight culture had significantly more chance for live birth (17).

We evaluated the effect of embryo quality on FET results. Veleva et al. showed the transfer of high quality embryos in FET cycles is the most important factor as regarded live birth. It was reported in cycles that no top quality embryo was cryopreserved; pregnancy rate is 13% in comparison with cycles that high quality embryo cryopreserved (31%) (18). Also, Niinimäki et al. reported the cumulative live birth is depended on the number of top quality embryos (19).

In the clinics that only top quality embryos were cryopreserved, the successes rate after FET was higher (1). Some references suggested that only top quality embryos with less than 10% fragmentation should cryopreserved (20). Gallardo et al. evaluated the implantation rate of vitrified embryos with 25% fragmentation and concluded unselecting of this embryos only leads to unwanted loss of embryos with acceptable implantation rate (16). In our experience grade C embryos had less survival rate than top quality embryos (with survival rate about 100%) (unpublished data). However, poor quality embryos that could survive after warming and specially show more cleavage after overnight culture had acceptable pregnancy rate, although this rate was less than good quality embryos. Therefore, the chance of patients that have only poor quality embryos for storage should not be ignored. Although, limitation of our study was characteristics of patients, such as body mass index, basic level of hormones and the oocytes number from the picked were not mentioned.

## Conclusion

In conclusion, overnight culture and confirmation of mitosis resumption is not essential for FET cycles in vitrification method.

## Figures and Tables

**Table 1:** Subject’s characteristics of FET* cycles in groups A** and B***

Variable	Group A** (*n* = 166)	Group B*** (*n* = 195)	*P*-value †
Female age (mean±SD)	30.89±4.73	30.48±4.78	0.975
Cause of infertility *N* (%)			
Female factor	71 (42.8)	88 (45.1)	0.619
Male factor	74 (44.6)	76 (39)	
Unknown	7 (4.2)	8 (4.1)	
Both	14 (8.4)	23 (11.8)	
No. of transferred embryo (mean±SD)	1.96±0.3	1.96±0.2	0.8
Embryo quality *N* (%)			
Grade A****	22 (13.3)	39 (20)	0.232
Grade B*****	122 (73.5)	133 (68.2)	
Grade C******	22 (13.3)	23 (11.8)	

* FET

** ET in the day of warming

*** ET in the one day after warming

**** With regular size blastomere, and no fragmentation

***** The embryos with uneven blastomeres and/or less than 10% fragmentation

****** The embryos with more than 10% fragmentation but no more than 25% of blastomeres

† The data were assessed using Chi-squared test and student’s *t*-test

**Table 2:** Comparison of clinical outcomes of FET* cycles between group A** and B***.

Variable	Group A** (*n* = 166)	Group B*** (*n* = 195)	*P*-value †
Chemical pregnancy *N* (%)	48 (28.9)	74 (37.9)	0.07
Clinical pregnancy *N* (%)	40 (24.1)	60 (30.8)	0.15
Live birth *N* (%)	37 (22.28)	38 (19.48)	0.8

* FET

** ET in the day of warming

*** ET in the one day after warming

† The data were assessed using student’s *t*-test

**Table 3:** Comparison of pregnancy rates between grades A*, B** and C embryos*** in FET**** cycles

Variable	Chemical pregnancy			Clinical pregnancy		
	
	Odds ratio	95%CI	*P*-value	Odds ratio	95%CI	*P*-value†
Age	0.99	0.94–1.94	0.8	0.99	0.95–1.04	0.91
Number of transferred embryos	0.79	0.4–1.56	0.51	1.19	0.77–1.83	0.41
Embryo grade	1.27	0.85–1.91	0.23	0.77	0.37–1.57	0.47

CI = confidence interval

* The embryos with regular size blastomere, and no fragmentation

** The embryos with uneven blastomeres and/or less than 10% fragmentation

*** The embryos with more than 10% but no more than 25% of blastomeres

**** FET

† Logistic regression
